# Therapeutic Inhibition of Pro-Inflammatory Signaling and Toxicity to Staphylococcal Enterotoxin B by a Synthetic Dimeric BB-Loop Mimetic of MyD88

**DOI:** 10.1371/journal.pone.0040773

**Published:** 2012-07-27

**Authors:** Teri L. Kissner, Gordon Ruthel, Shahabuddin Alam, Enrique Mann, Dariush Ajami, Mitra Rebek, Eileen Larkin, Stefan Fernandez, Robert G. Ulrich, Sun Ping, David S. Waugh, Julius Rebek, Kamal U. Saikh

**Affiliations:** 1 Department of Immunology, United States Army Medical Research Institute of Infectious Diseases, Frederick, Maryland, United States of America; 2 Department of Chemistry, The Skaggs Institute for Chemical Biology, The Scripps Research Institute, La Jolla, California, United States of America; 3 Macromolecular Crystallography Laboratory, National Cancer Institute at Frederick, Frederick, Maryland, United States of America; National Council of Sciences (CONICET), Argentina

## Abstract

Staphylococcal enterotoxin B (SEB) exposure triggers an exaggerated pro-inflammatory cytokine response that often leads to toxic shock syndrome (TSS) associated with organ failure and death. MyD88 mediates pro-inflammatory cytokine signaling induced by SEB exposure and MyD88^−/−^ mice are resistant to SEB intoxication, suggesting that MyD88 may be a potential target for therapeutic intervention. We targeted the BB loop region of the Toll/IL-1 receptor (TIR) domain of MyD88 to develop small-molecule therapeutics. Here, we report that a synthetic compound (EM-163), mimic to dimeric form of BB-loop of MyD88 attenuated tumor necrosis factor (TNF)- α, interferon (IFN)-γ, interleukin (IL)-1β, IL-2 and IL-6 production in human primary cells, whether administered pre- or post-SEB exposure. Results from a direct binding assay, and from MyD88 co-transfection/co-immunoprecipitation experiments, suggest that EM-163 inhibits TIR-TIR domain interaction. Additional results indicate that EM-163 prevents MyD88 from mediating downstream signaling. In an NF-kB-driven reporter assay of lipopolysaccharide-stimulated MyD88 signaling, EM-163 demonstrated a dose-dependent inhibition of reporter activity as well as TNF-α and IL-1β production. Importantly, administration of EM-163 pre- or post exposure to a lethal dose of SEB abrogated pro-inflammatory cytokine responses and protected mice from toxic shock-induced death. Taken together, our results suggest that EM-163 exhibits a potential for therapeutic use against SEB intoxication.

## Introduction

The profound clinical consequences of staphylococcal enterotoxin B (SEB)-induced toxicity are known to stem from an excessive pro-inflammatory cytokine response that often leads to toxic shock syndrome (TSS) associated with organ failure and death. Strong pro-inflammatory responses by antigen-presenting cells (APCs) and T cells are triggered by the binding of SEB to MHC class II molecules on APCs and subsequent cross-linking to T-cell receptors (TCR) [1]–[5]. SEB is listed by the Centers for Disease Control and Prevention (CDC) as a Category B select agent because of its potential use as an aerosolized biological weapon. There are currently no small-molecule therapeutics available to treat SEB exposure, making the development of such medical countermeasures an important goal.

In a mouse model, the biological effects of SEB are potentiated by lipopolysaccharide (LPS), a bacterial factor that binds to toll-like receptor 4 (TLR4) on the surface of cells. SEB and LPS synergistically amplify pro-inflammatory cytokine production, leading to severe toxicity [6]–[7]. Consequently, both superantigenic exotoxins (SEs) and bacterial LPS (endotoxin) have been implicated in the pathogenesis of TSS, supported by their identification in the bloodstream of critically ill patients with septic shock [8]–[9]. Results from our laboratory demonstrated that myeloid differentiation protein 88 (MyD88) gene knockout (MyD88^−/−^) mice were resistant to lethal SEA or SEB challenge and showed a concomitant reduction in serum levels of pro-inflammatory cytokines [10]–[11]. We also reported that the binding of SEA or SEB to MHC class II activates MyD88- mediated pro-inflammatory cytokine signaling in human primary cells [12]. Consistent with our results, a recent report indicated that deficiency in MHC class II resulted in impaired TLR-triggered production of pro-inflammatory cytokines and protected mice from an otherwise lethal challenge with TLR ligands and live Gram-negative bacteria [13]. This study also concluded that both the TLR-and MHC –mediated responses engage MyD88 [14].

MyD88 is an adaptor protein that functions to recruit signaling proteins to members of the Toll-like and interleukin-1 receptor (TLR/IL-1R) [15]–[16] family as well as the IFN -γ receptor [17]. Activation of the MyD88-mediated pro-inflammatory signaling pathway by this class of receptors is very important for several aspects of host defense. Because of the critical role of MyD88 signaling, mice deficient in MyD88 have profoundly impaired innate immune responses and are susceptible to a wide range of infectious diseases [18]. However, in regards to innate immune-response regulation, MyD88 signaling performs a delicate balancing act. Perturbed regulation or excessive stimulation of the innate immune system can trigger, inflammatory signaling that can spiral out of control and lead to profound clinical syndrome [18]. For example, excess of MyD88-mediated signaling can lead to severe pathological consequences such as toxic shock syndrome (TSS) and sepsis. However, the crucial role of MyD88 in these disorders also provides a target for therapeutic intervention.

Earlier results from our laboratory demonstrated that a synthetic mimetic of the BB-loop in the TIR domain of MyD88 (Compound 1) attenuated SEB-induced pro-inflammatory cytokine production in human primary cells and increased survivability of mice from toxic shock-induced death after a lethal SEB challenge [19]. It is known that the BB-loop region acts as the mediator of the homo- (adaptor-adaptor) and hetero- (receptor-adaptor) dimerization that is necessary for the function of TIR domains to induce MyD88-mediated signaling [15], [16]. Seeking to improve the efficacy of Compound 1 as an inhibitor of MyD88 signaling, we synthesized a dimeric molecule in which two Compound 1 moieties were covalently linked together, reasoning that the dimeric compound would be a more potent inhibitor of protein-protein interactions. This molecule, EM-163, was tested in primary cultures of human mononuclear cells (MNCs) for inhibition of cytokine release associated with exposure to SEB. Our results provide evidence that, by targeting MyD88, EM-163 inhibited SEB-induced inflammatory cytokine production in human primary cells. Importantly, EM-163 abrogated pro-inflammatory cytokine response *in vivo* and completely protected mice from toxic shock-induced death regardless of whether it was administered pre- or post-exposure to a lethal SEB challenge.

## Materials and Methods

### Reagents

Staphylococcal enterotoxin B (SEB) and SEA was purchased from Porton Down, Inc. (Salisbury, UK) and stored at −50°C. SEB or SEA was endotoxin free and prepared under GMP conditions. *Escherichia coli* LPS (055:B5) was purchased from Sigma-Aldrich (Saint Louis, MO). Pooled human AB sera were obtained from Pel-Freez (Brown Deer, WI). The BD™ cytometric bead array (CBA) human Th1/Th2 and inflammatory cytokine kit was purchased from BD Biosciences (San Diego, CA). The Meso Scale Discovery (MSD) multi spot array ultra-sensitive cytokine assay kit was purchased from MSD (Gaithersburg, MD). Magnetic bead-conjugated anti-CD14 and anti-CD3 mAbs were obtained from Miltenyi Biotech Inc. (Auburn, CA). Ficoll-Hypaque was purchased from GE Healthcare Biosciences (Piscataway, NJ). Primary anti-MyD88 antibody was obtained from AnaSpec, Inc., (San Jose, CA) and Alexis Biochemicals (San Diego, CA). Anti-β-actin antibody was purchased from Cell Signaling technology (Danvers, MA). Plasmids expressing HLA-DRα/β were a kind gift from Dr. Robert Ulrich (USAMRIID). Plasmid 12287 (pCMV-HA-MyD88) and plasmid 13093 (MyD88-flag) were purchased through an MTA agreement with Addgene (Cambridge, MA). The HEK 293 T cell line was obtained from the ATCC (Manassas, VA). MyD88 KO HEK293 cell line (HEK293-I3A) was a kind gift from G. Stark (Dept. of Molecular Genetics, Lerner Research Institute, Cleveland Clinic, OH). The transfection reagent lipofectamine was purchased from Invitrogen (Carlsbad, CA). Vectashield mounting medium containing 4-,6-diamidino-2-phenylindole (DAPI) was purchased from Vector Laboratories (Burlingame,CA).

### Mice

Pathogen-free, 6–8 weeks old BALB/c and C57BL/6 mice were obtained from Charles River (NCI-Frederick, Frederick, MD).

### Cell Isolation and Purification

Peripheral blood mononuclear cells (MNC) were obtained from consenting healthy donors in accordance with an Institutional Review Board-approved research donor protocol. MNCs were isolated by standard density gradient centrifugation with Ficoll-Hypaque, harvested from the interface, washed, and re-suspended in RPMI 1640 medium. Monocytes (CD14^+^) and T (CD3^+^) cells were isolated as previously described [19]. Isolated cell populations had >98% purity.

### Cytokine Analysis

Cell cultures were incubated (37°C, 5% CO_2_) for 16 h. Cytokines in culture supernatants were measured by a CBA kit using captured beads coated with antibodies specific for cytokines. Flow cytometry was performed as described elsewhere [20]. Cytokine measurement was confirmed by dilution of culture supernatant using Inflammation and Th1/Th2 CBA kits by acquiring 1800 beads. We also used a Meso Scale Discovery (MSD) multi spot array ultra sensitive cytokine assay kit to measure cytokines in culture supernatants (according to the manufacturer’s protocol) as described [19].

### Western Blot Analysis

The transfected cells were chilled on ice for 5 min before being pelleted into fresh 1.5 ml centrifuge tubes. Membrane and cytoplasm separation was done by suspending the pellets in 50 µl of lysis buffer (Active Motif) in the presence of DTT, protease inhibitors and phosphatase inhibitors and incubated on ice for 30–60 min. The membrane fraction was collected by centrifuging the lysates at 14000×*g* for 20 min. The supernatant contained the cytoplasmic fraction. Samples containing 10 µg of total cytoplasmic proteins were separated by gel electrophoresis and transferred to nitrocellulose membranes. The membranes were blocked overnight in 1× Tris-buffered saline (TBS) containing 0.1% Tween-20 and 3% bovine serum albumin at 4 ^o^C. The membranes were washed extensively with 1× TBS buffer and then probed with anti-MyD88 polyclonal antibody followed by horseradish peroxide-conjugated secondary antibody (goat anti-rabbit). After additional rinsing with 1× TBS buffer, the membranes were exposed to a chemiluminescent substrate in the presence of hydrogen peroxide, using Immun-Star WesternC Chemiluminescent kit (BioRad). A VersaDoc Model 4000 (BioRad) imaging system was used to capture the image.

### Intracellular Staining and Confocal Microscopy

Freshly isolated monocytes (CD14^+^) adhered to sterile culture slides (Corning Glass, Corning, NY) were incubated with either SEB (200 ng/ml), SEB+EM-163, or kept untreated in RPMI 1640 medium. Cells were gently washed with PBS (pH 7.4, 4°C) and then fixed with 1% paraformaldehyde (Tousimis Research, Rockville, MD) plus 0.1% glutaraldehyde (Sigma-Aldrich) in PBS (5 min, 20°C). The fixed cells were washed with PBS containing 0.5% bovine serum albumin (BSA) and incubated (30 min, 20°C) in a permeabilization solution (Becton Dickinson). Cells were then washed and incubated (15 min, 20°C) with PBS containing 5% BSA to block non-specific antibody binding. The cells were then incubated with primary MyD88 antibody for 1 h, washed in PBS containing 0.5% BSA, then incubated with Alexa 568-conjugated goat anti-rabbit secondary antibody. The cells were counterstained with DAPI to detect cell nuclei. Labeled cells were mounted on glass slides using fluoromount and covered with glass cover slips. Labeled cells were imaged using a Leica (Mannheim, Germany) TCS SP5 laser scanning confocal system. Green and red fluorescence were visualized using 488-nm and 568 nm wavelength laser excitation, respectively, and DAPI images were collected using two-photon excitation from a Ti/sapphire laser tuned to 780 nm.

### Synthesis of Compound EM-163

Initially, Compound 1 was used to synthesize EM-110 [20], as follows. Briefly, to a stirred solution of 2,5-pyridine dicarboxylic acid (52 mg; 0.3 mmol) in CH_2_Cl_2_ (0.5 ml) and dimethylformamide (10 µl), thionyl chloride (0.4 ml) was added drop-wise at 0°C. The mixture was then heated at 40°C for 1 h, and the solvent was removed under reduced pressure. The diacid chloride obtained was added to a solution of amine **2** (200 mg; 0.69 mmol) and Et3N (97 µl; 0.69 mmol) in dry CH_2_Cl_2_ (10 ml). The reaction mixture was stirred at ambient temperature overnight, the solvent was evaporated, and the residue was purified by silica gel chromatography (hexane/AcOEt mixtures) yielding compound **4** (36 mg; 17% yield) as a white solid. ^1^H NMR: (600 MHz, CDCl3) δ 8.39 (s, 1H), 7.69 (d, *J*  = 8.1 Hz, 1H), 7.55 (d, *J*  =  8.1 Hz, 1H), 7.20 (m, 4H), 7.11 (m, 2H), 6.94 (m, 2H), 6.86 (m, 2H), 5.21 (m, 2H), 3.70 (m, 4H), 3.59 (m, 2H), 3.43 (m, 2H), 3.31 (m, 4H), 2.51 (m, 2H), 2.40 (m, 2H), 2.29 (m, 2H), 1.98 (m, 4H), 1.90 (m, 4H), 1.68 (m, 2H), 1.54 (m, 2H), 1.04 (m, 12H); ^13^C NMR: (150 MHz, CDCl3) δ169.6, 169.0, 168.5, 168.4, 155.6, 145.8, 141.4, 140.7, 135.1, 133.8, 128.9 (2C), 128.8 (2C), 128.5 (2C), 128.3 (2C), 126.4, 126.2, 123.6, 60.9, 60.4, 47.2, 47.0, 46.5 (2C), 45.2, 44.8, 33.6, 33.4, 31.8, 31.7, 28.3 (2C), 26.6 (2C), 24.6 (2C), 20.5, 20.3, 18.9, 18.8; MS: (ESI-TOF) MH^+^ calculated:708.4488, found: 708.4461.

**Figure 1:**
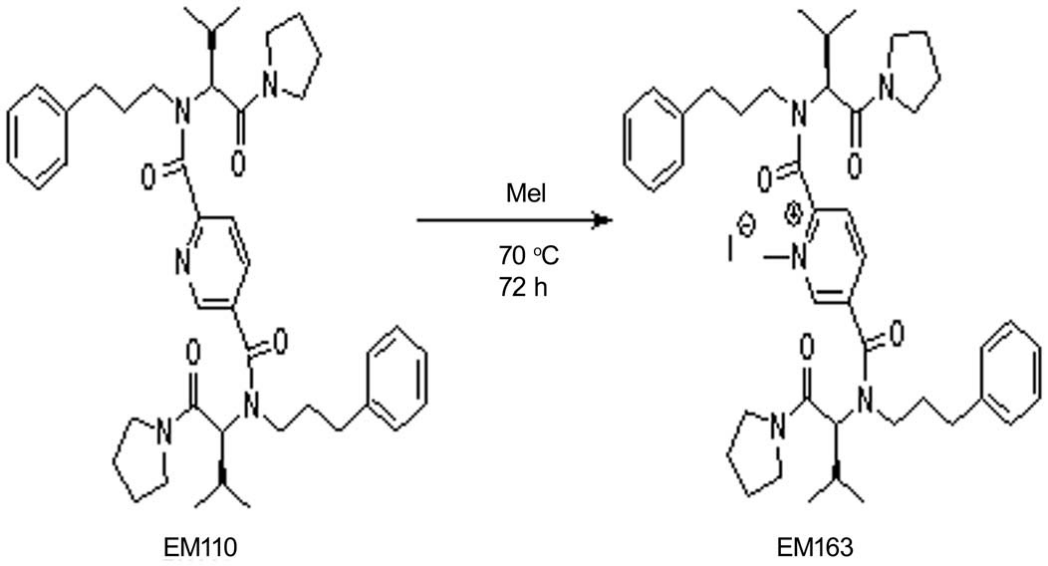
Synthesize of compound EM-163. EM-163 was synthesized from compound EM-110 as described in Materials and Methods.

EM-163 was synthesized from compound EM-110 [20] as shown in Figure 1. Compound EM-110 (600 mg, 0.85 mmol) and methyl iodide (10 ml) were stirred at 70°C for 72 h. The excess methyl iodide was then eliminated under a stream of nitrogen and the residue was purified by column chromatography (AcOEt/MeOH, 95∶5) to afford compound EM163 (380 mg, 53%) as a orange solid. 1H NMR (600 MHz, CD_3_CN, 50°C, mixture of conformers) δ 9.70 (bs, 1H), 8.58 (bs, 1H), 8.16 (bs, 1H). 7.25–6.90 (m, 10 H), 5.07 (m, 2H), 4.48 (s, 3H), 3.62 (m, 4H), 3.53 (m, 2H), 3.41 (m, 2H), 3.32 (m, 4H), 2.55 (m, 2H), 2.45 (m, 2H), 2.37 (m, 2H), 1.95 (m, 4H), 1.86–1.70(m, 8H), 1.00–0.85 (m, 12H). MS: (ESI-TOF), M^+^ calculated: 722.4845, found: 722.4837.

### Expression of MyD88 Protein

The open reading frame encoding the TIR domain of human MyD88 (residues 157–296) was amplified from pCDNA3-MyD88-GFP (Addgene plasmid 13026, Addgene, Cambridge, MA, USA) and inserted into pDONR201 (Invitrogen, Carlsbad, CA,). The gene was sequence verified and then inserted into the destination vector pDEST-HisMBP [21] by Gateway recombinatorial cloning to generate expression vector pPS2218.

The recombinant His-MBP-MyD88(157–296) fusion protein was expressed in *E. coli* BL21(DE3) Codon Plus-RIL cells (Stratagene, La Jolla, CA), which were grown in Luria broth and induced at mid-log phase with 1 mM IPTG for 4 h at 30°C. The cells were harvested by centrifugation at 4°C and frozen at −80°C until use. The cell pellet was resuspended in 50 mM sodium phosphate pH 7.1, 150 mM NaCl, 5% glycerol, 25 mM imidazole, and then the cells were disrupted using an APV Model G1000 homogenizer (Invensys, Roholmsvej, Denmark). The lysate was centrifuged at 15,000 rpm at 4°C, filtered, and the fusion protein was then purified by immobilized metal affinity chromatography (IMAC) as described [22]. Fractions containing the fusion protein were pooled, cleaved overnight with hexahistidine-tagged TEV protease, and then subjected to another round of IMAC as described [22]. The flow-through fractions were pooled, concentrated to 5 ml and applied to a 320 ml XK26/60 Sephacryl S-100 gel filtration column (GE Healthcare, Piscataway, NJ) equilibrated in 25 mM 2-(*N*-morpholino) ethanesulfonic acid (MES) pH 6.3, 150 mM NaCl, and 2 mM Tris(2-carboxyethyl) phosphine hydrochloride (TCEP). The peak fractions corresponding to MyD88 (157–296) were pooled and concentrated to approximately 1 mg/ml.

### Microarray-based Binding Assay

A microarray-based binding assay was developed and optimized to measure the binding of compound EM-163 to MyD88. Nitrocellulose-coated slides (PATH slides, Gentel Biosciences, Madison, WI) were spotted in quadruplicate with anti-MyD88 antibody or MyD88 monomer using the ArrayJet Marathon printer (Roslin, Scotland, UK). Slides were blocked overnight (∼16 h) at 4 ^o^C with blocking buffer (50 mM Hepes, 0.08% triton X-100, 50% glycerol, 140 mM NaCl and 3% BSA). Recombinant MyD88 was bio-tinylated using the EZ Link Biotin BMCC kit from Pierce (Cat# PI-21900, Pittsburg PA). Biotinlyated MyD88 (25 µg/ml) was pre-incubated with and without compound (100 µg/ml and 50 µg/ml) for 1 h on ice and then diluted with probing buffer (50 mM Hepes, 0.1%Tween, 3% human antibody serum). MyD88 samples and a probing buffer only control were added to designated wells on the microarray slide for 2 h at room temperature. Slides were then washed 6 times for 5 min with washing buffer (50 mM Hepes, 0.2%Tween, 3% human antibody serum). Streptavidin labeled with alexa fluor 647 (1∶1000, Invitrogen, Carlsbad CA) was then added for 1 h at room temperature to detect MyD88 binding interaction. Slides were again washed as before and then rinsed with distilled water for 10 sec, dried and analyzed using the Genepix 4000B (Molecular Devices, Sunnyvale, CA).

### Cell Culture and Transfections

Human embryonic kidney (HEK) 293T cells were cultured in EMEM, supplemented with 10% fetal bovine serum (FBS) (Invitrogen, Carlsbad, CA), and grown in a 37°C humidified atmosphere of 5% CO_2_. For co-immunoprecipitation of MyD88-Flag/HA-MyD88, HEK cells were cultured in 6-well plates and transfected by lipofectamine 2000 (Invitrogen) method with 4–5 µg of the appropriate plasmids according to the manufacturer’s instructions. The MyD88 mimetic EM-163 was added to the medium 6 h after transfection. For SEB-induced cytokine inhibition by EM-163, HEK 293 cells were transfected with plasmids expressing HLA-DR (α/β) and Flag-MyD88. Six h after transfection, cells were cultured with SEB in the presence or absence of EM-163. Culture supernatants were collected after 24 h for measuring cytokine production.

### Co-immunoprecipitation Assay

HEK293T cells (transfected or Mock) were collected 48 h after transfection, washed with 2 ml of ice-cold PBS, and lysed in 80 µl of buffer [50 mM HEPES, pH7.4]. Cells were pelleted by centrifugation at 10, 000 x g for 10 min at 4°C, and cytosolic fractions were collected for immunoprecipitation. Cell extracts (1 mg total proteins) were incubated with 2 µg of mouse anti –Flag M2 conjugated with agarose attached to magnetic beads (Sigma-Aldrich) for 16 h under constant shaking at 4°C. Agarose bead-bound immunocomplexes were separated by a magnetic separator, washed three times, and eluted in SDS-PAGE sample buffer for western blot analysis.

### Secreted Alkaline Phosphatase (SEAP) Assay

TLR4/MD-2/NF-kB/SEAPorter HEK 293 cells (5×10^5^ cells/ml/well) were cultured with LPS (1 µg/ml) or poly IC (1 µg/ml), with varying concentrations of EM-163 in a 24-well plate and incubated at 37°C for 16 h. The culture supernatant was collected and centrifuged to remove any cell debris. The Great EscAPe SEAP assay from Clonetech was used to determine the amount of alkaline phosphatase secreted into the supernatant. A 1× dilution buffer was prepared from a 5× stock solution and 75 µl of the 1× dilution buffer was mixed with 25 µl of the supernatant, incubated for 30 min at 65 ^o^C to inactivate endogenous alkaline phosphatase. The samples were placed on ice for 3 min and then equilibrated at room temperature. SEAP substrate solution (100 µl) was added to each sample and read at 10 min intervals using a chemiluminescence reader.

### Statistical Analysis

The SAS program v9.2 (Cary, NC, USA) was used for statistical analysis. The planned analyses using T-tests with step-down Bonferroni adjustment to compare geometric mean NF-kB activation and cytokine levels between groups were undertaken for determining statistical significance. Kaplan- Meier survival analysis (dependent variables: survival status, time to death) and log-rank test to compare survival curves (with step-down Bonferroni adjustment for pair wise comparisons) among groups were performed.

## Results

### Design and Synthesis of a Dimeric BB-loop Mimetic of MyD88 (EM-163)

**Figure 2:**
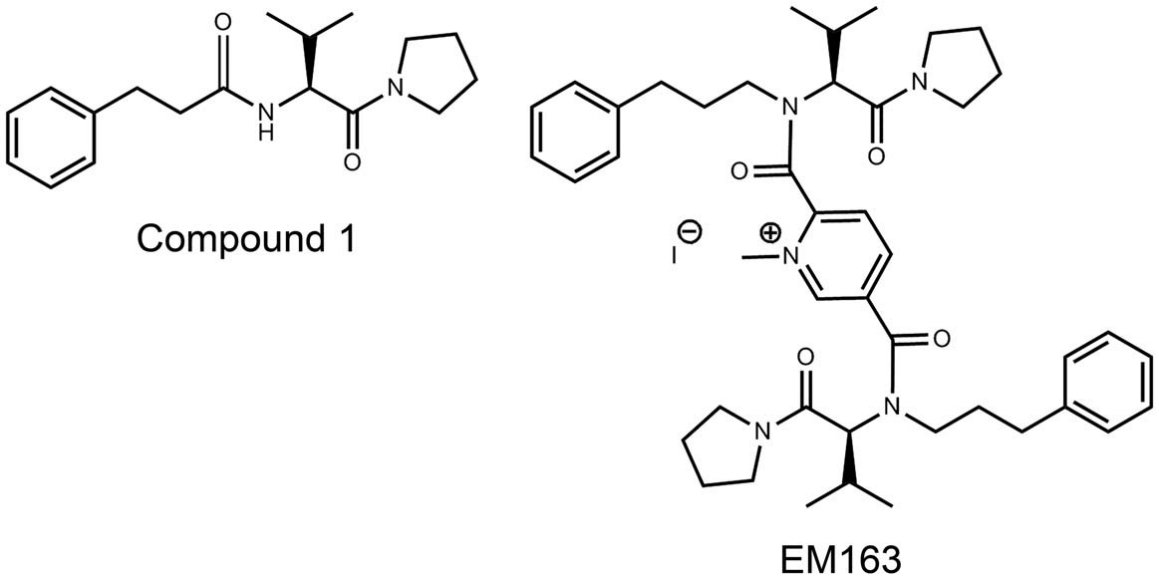
Structure of monomeric (Compound1) and dimeric (EM-163) mimetics of the BB-loop in the TIR domain of MyD88.

Several structural and mutational studies have pointed to the BB-, DD- and EE-loop regions as mediators of the homo- or hetero-dimerization function of TIR domains in bacteria and mammals [23]–[26]. However, neither the homotypic nor heterotypic interactions between TIR domains of receptors and adaptors are well understood [27]. Our earlier results indicated that a synthetic mimetic, Compound 1, modeled on a tripeptide sequence of the BB-loop [(F/Y)-(V/L/I)-(P/G)] of the TIR domain, showed promise in attenuating SEB as well as TSST1-induced pro-inflammatory cytokine production in human primary cell culture [19]. Because of the importance of homotypic and heterotypic interactions in MyD88 signaling, we hypothesized that a BB-loop mimetic with a dimeric structure might be more effective in blocking MyD88-signaling. Therefore, in an attempt to improve specificity and therapeutic efficacy, a dimeric compound EM-110 was synthesized based on the original structure of Compound 1 [20]. Further chemical modification of EM-110 by treatment with methyl iodide led to the synthesis of a quaternary ammonium salt: the stable crystalline solid compound EM-163 (Figure 2).

### EM-163 Binds to TIR Domain and Inhibits TIR-TIR Homotypic Interaction

**Figure 3:**
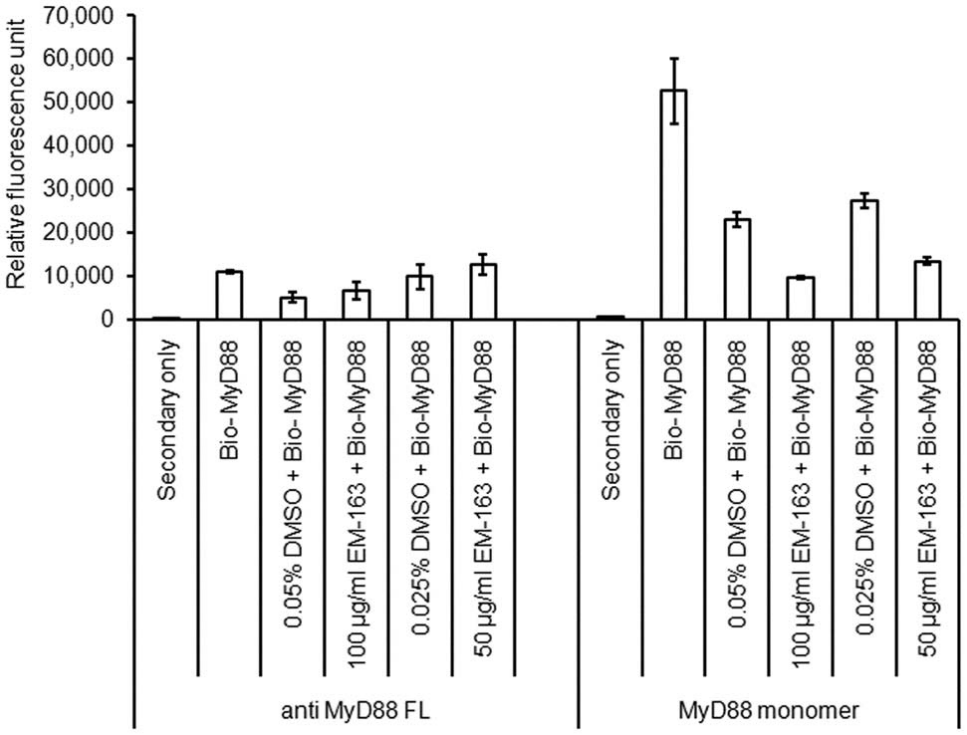
EM-163 inhibits TIR-TIR interaction. A microarray-based binding assay was optimized to measure compound EM-163 binding to MyD88. Nitrocellulose coated slides were spotted in quadruplicate with anti-MyD88 antibody and MyD88 TIR domain monomer proteins using the ArrayJet Marathon printer. Biotinlyated MyD88 (25 µg/ml) was pre-incubated with compound (100 µg/ml and 50 µg/ml), dimethylsulfoxide as control, or left without pre-incubation. Streptavidin- labeled with alexa fluor 647, was added for 1 h at room temperture to detect MyD88 binding interaction. Slides analyzed using the Genepix 4000B. Data represent four similar experiments.

TIR domain interaction of MyD88 is required for its recruitment to the receptor complex, a critical step for MyD88-mediated pro-inflammatory signaling via the ensuing activation of the downstream kinases IRAK1 and IRAK4. To determine if EM-163 interferes with TIR-TIR domain interaction, we examined the effect of EM-163 binding to recombinant TIR domain MyD88 protein in a microarray-based binding assay. We expressed and purified the recombinant MyD88 TIR domain (amino acids 157–296) as a histidine-tag GST fusion protein. In SDS-PAGE a molecular mass of 16.4 kDa of the MyD88 TIR domain protein was observed, as expected. A MyD88 binding array was developed and optimized to detect inhibition of the TIR–TIR domain interaction. The results shown in Figure 3 indicate interaction of TIR domain protein (monomer) coated on chips with biotinylated TIR protein. Pre-incubation of immobilized TIR domain protein with EM-163 resulted in a dose-dependent reduction of the signal, suggesting that EM-163 binding to TIR domain protein inhibited the TIR–TIR domain interaction (Figure 3).

### Increase in Accumulation of MyD88 in the Presence of EM-163

**Figure 4:**
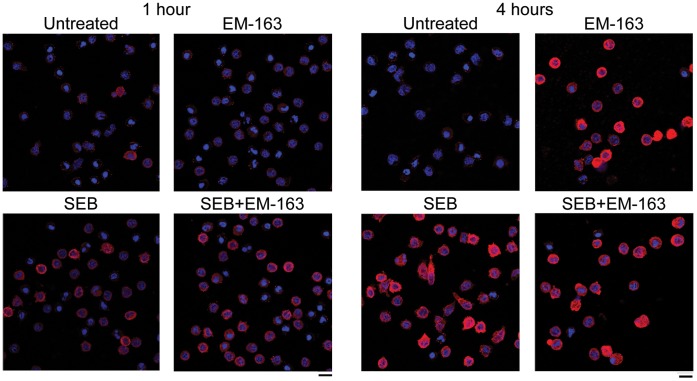
SEB stimulation of human monocytes in the presence of EM-163 leads to intracellular accumulation of MyD88. Increased accumulation of MyD88 in CD14^+^ monocytes treated with SEB in the presence of EM-163 at 1 h and 4 h. Confocal images show expression of nuclei (blue) and intracellular MyD88 (red) proteins in CD14^+^ monocytes. Monocytes were untreated or treated with SEB, EM-163 or SEB plus EM-163 (250 µM). Cell nuclei were labeled with DAPI (blue). Scale bar  = 10 µm. Data reperesent three similar experiments with different donors.

Our earlier results demonstrated that SEB stimulation induces *de novo* synthesis of MyD88 in primary cells compared to non-stimulated cells [12], [19]. We examined the effects of EM-163 on levels of MyD88 protein in SEB-stimulated and unstimulated primary monocytes. Purified monocytes were treated with SEB in the presence or absence of EM-163 and levels of MyD88 were analyzed by confocal microscopy. Consistent with the previously reported up-regulation of MyD88, monocytes stimulated with SEB caused an increase in intracellular accumulation of MyD88 (Figure 4). An increase in MyD88 levels was likewise seen with SEB stimulation in the presence of EM-163 both at 1 hr and 4 hr. However, an increase in MyD88 levels was observed in some unstimulated cells after treatment with EM-163 only at 4 hr. Similar effects of EM-163 on T cells (CD3^+^MyD88^+^) were observed when examined by dual color flow cytometric analysis (data not shown). A possible explanation for these results is that EM-163, having two BB-loop mimetic groups, capable of binding to the basal MyD88 molecules in the cells and stabilized to allow accumulation within the cell.

**Figure 5:**
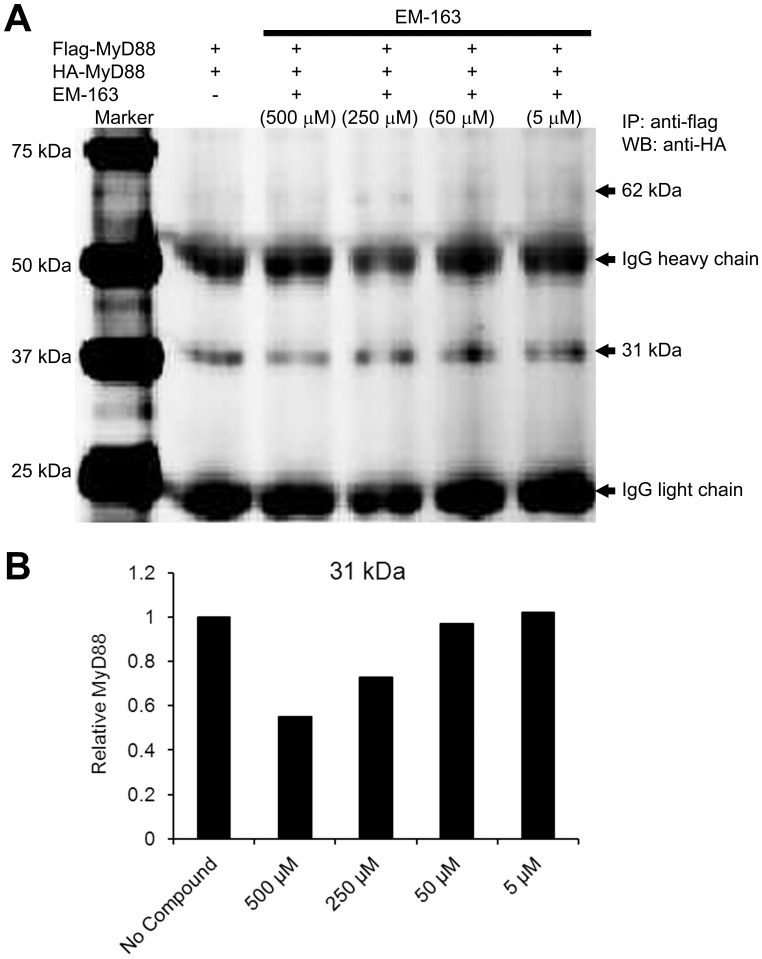
EM-163 targets newly expressed MyD88 and stabilizes the MyD88 complex. HEK 293T cells were co-transfected with plasmids MyD88- Flag or pCMV-HA-MyD88. Seven hours after transfection, cells were incubated for 13 h with or without −163 (500 µM to 5 µM). (**A**), Cell extracts were immunoprecipitated (IP) with anti-Flag antibody, and immune-precipitated proteins were analyzed in Western blot with anti-HA. (**B**) Densitometry analysis of the results shown in (**A**). The results are representative of three independent experiments.

At low concentrations, EM-163 would be expected to be more likely to bind two MyD88 monomers than at saturating concentrations, where there would be enough EM-163 molecules to bind MyD88 monomer at a 1∶1 ratio. We therefore examined if this prediction would be borne out experimentally. In addition, because SEB stimulation induces up regulation of MyD88, we wished to test if newly synthesized MyD88 would be targeted by EM-163. To this end, HEK 293T cells were co-transfected with plasmids pCMV-HA-MyD88 and MyD88-Flag and 6 h later were treated with varying concentrations of EM-163. In a co-immunoprecipitation assay using anti-Flag antibody, followed by SDS-PAGE and immunoblot analysis with anti-HA antibody, EM-163 treatment inhibited the presence of 62 kDa protein hence, increased the accumulation of 31 kDa proteins in a dose-dependent manner (Figure 5A). While controls, i.e., no compound treated pCMV-HA-MyD88 and MyD88-Flag transfected cells (Figure 5A) or only DMSO treated cells had no inhibitory effect of 62 kDa (data not shown), because more accumulation of 31 kDa band was observed compared to EM-163 treatment. Using the same experimental set up as for the results shown in Figure 5A, the presence of 31 kDa MyD88 was confirmed by reprobing with anti-MyD88 antibody (data not shown). These results suggest that EM-163 prevented the association of Flag-MyD88 -HA-MyD88 complex (Figure 5B) in a dose-dependent manner by targeting to newly synthesized MyD88. To further confirm that newly expressed MyD88 are targeted by EM-163, we performed an additional experiment using MyD88 KO HEK293 cell line (HEK293-I3A). Similar to results shown in Figure 5, co-immunoprecipitation assay using anti-Flag antibody, followed by SDS-PAGE and immunoblot analysis demonstrated that EM-163 treatment inhibited in a dose dependent mannar MyD88 dimer, and thereby, allowed accumulation of 31 kDa MyD88 (Figure S1). It may likely be that the monomeric form of the MyD88 TIR domain, with its exposed BB loop, is the molecular target of EM-163. Thus, EM-163 appeared able to target newly expressed MyD88 and to prevent MyD88 complexes. However, it was important to ascertain if the interaction of EM-163 with MyD88 could inhibit downstream signaling.

### EM-163 Blocks MyD88-mediated Signaling

**Figure 6:**
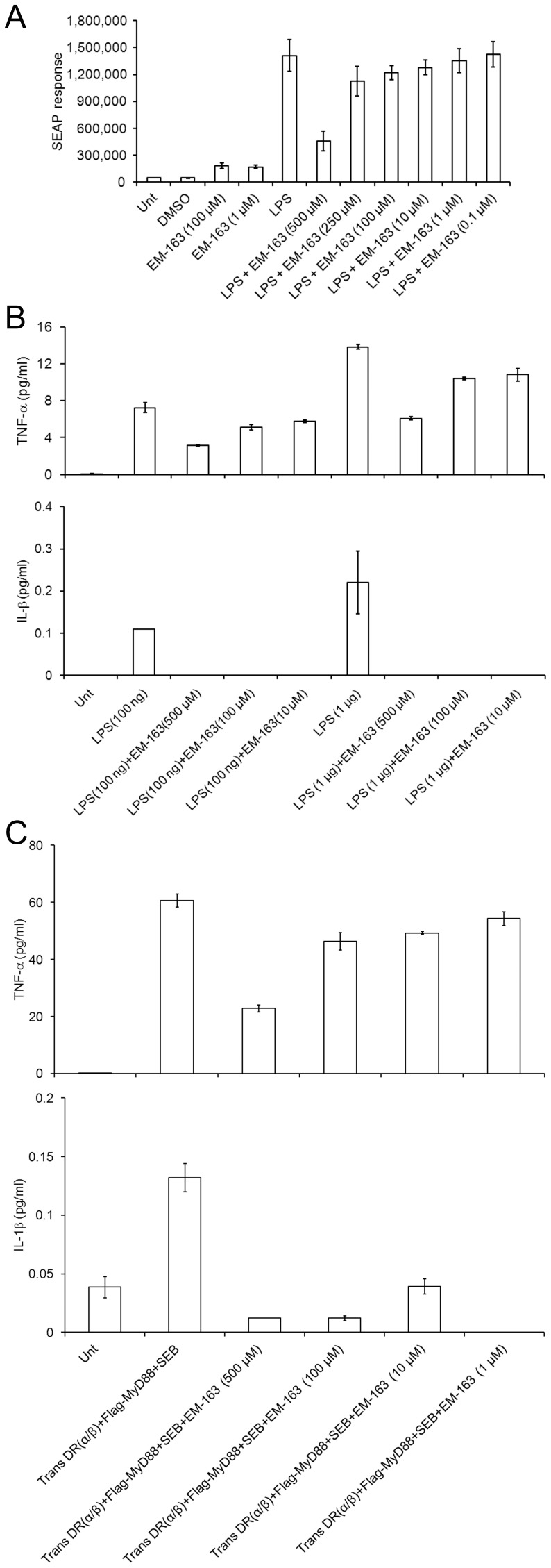
EM-163 inhibits MyD88-mediated signaling and cytokine production. EM-163 inhibition was measured by monitoring LPS-induced SEAP activity via a MyD88-mediated NF-kB driven signaling pathway. HEK 293 stable transfected cell line (TLR4-MD2-NF-kB-SEAP) was activated with LPS (TLR-4 ligand) and treated with varying concentrations of EM-163 (1 mM to 50 µM). Culture supernatants were tested for SEAP activity and compared to levels in the absence of EM-163. (**A**) Inhibition of SEAP reporter activity, (**B**) inhibition of TNF-α and IL-1β production, *(* **C**) Inhibition of TNF-α and IL-1β production by EM-163 after SEB stimulation of HEK 293 cells transfected with HLA-DR (α/β) and FLAG-MyD88. Results represent three experiments.

To determine if the inhibition of TIR-TIR interaction demonstrated above had an effect on signaling, we utilized a cell-based reporter assay. A stable co-transfected (TLR4-MD2-NF-kB/SEAPorter™) HEK 293 cell line was used to detect a MyD88-mediated NF-kB driven SEAP reporter response induced by LPS. As shown in Figure 6A, EM-163 inhibited the LPS-induced TLR4- MD2-MyD88-NF-kB-SEAP response in a dose-dependent manner. In contrast, TLR3 ligand poly IC stimulation, which does not utilize MyD88, had no effect on SEAP response (data not shown). In addition to inhibition of the SEAP response to LPS stimulation, EM-163 also blocked LPS-induced TNF-α and IL-1β production by these cells in a dose-dependent manner (Figure 6B **)**. We also confirmed that EM-163 was capable of inhibiting cytokine production stimulated by SEB in a similar HEK 293 cell model. HEK 293 cells were co-transfected with HLA-DR (α/β) and Flag-tagged MyD88 plasmids and stimulated with SEB in the presence or absence of EM-163. Culture supernatants were then tested for the presence of TNF-α and IL-1β. Results shown in Figure 6C demonstrate SEB-stimulated production of TNF-α and IL-1β by HEK 293 cells expressing HLA-DR and MyD88, and its inhibition by EM-163. These results clearly demonstrate that MHC class II-linked activation of MyD88 signaling [12] was also inhibited by EM-163.

### EM-163 Inhibits SEB-induced TNF-α, IFN-γ, IL-1β, IL-2 and IL-6 Production in Human Primary Cells

**Figure 7:**
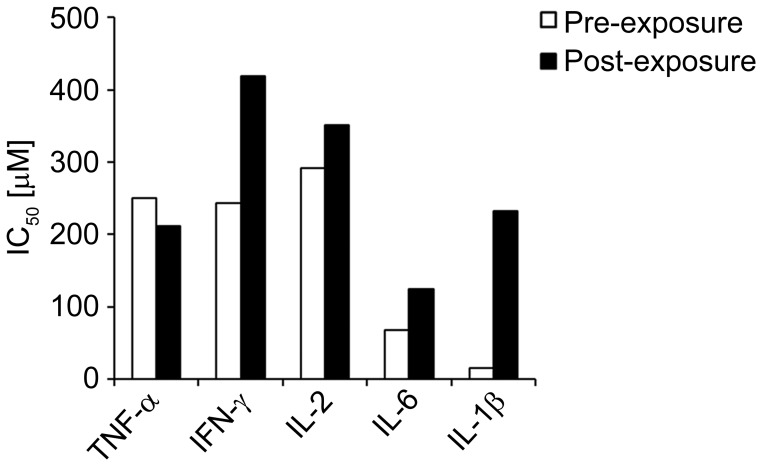
Pre or post exposure treatment of EM-163 inhibits TNF-α, IFN-γ, IL-2 and IL-1β production. Human MNCs (5×10^5^/well) were treated with EM-163 for 30 min before (pre-exposure) or 30 min after (post-exposure) being exposed to SEB(200 ng/ml) and varying concentrations of EM-163 (1 mM, 500 µM, 100 µM and 10 µM). Culture supernatants were collected after 16 h and analyzed for cytokine release using a MSD assay. The IC_50_ value was calculated as the concentration required for inhibition of cytokine production by 50% relative to the control. Data represent five experiments.

The results above indicated that EM-163 inhibits downstream signaling by targeting MyD88 in a transfected HEK 293 cell line, so next we asked if the change in signaling would likewise lead to a decrease in cytokine production in cultured human primary cells. To determine therapeutic potential, it was also important to examine the efficacy of EM-163 when administered after SEB exposure. To assess the inhibitory effects of EM-163 on SEB-induced cytokine production in primary culture, MNCs were treated with EM-163 for 30 min before (pre-exposure) or 30 min after (post-exposure) being exposed to SEB. Culture supernatants were collected after 16 h and analyzed for cytokines. As shown in Figure 7, pre- or post-exposure treatment with EM-163 inhibited SEB-induced TNF-α, IFN-γ, IL-1β, IL-2 and IL-6 production (IC_50_ 15 µM to 400 µM), although EM-163 was generally more effective (had a lower IC_50_) when applied pre-SEB exposure compared to post-exposure treatment. As predicted, EM-163 was more effective than Compound 1(IC_50_ 200 µM to 1.2 mM) [19]. The cytokine inhibitory effect of EM-163 on cultured cells was consistent among populations of MNCs isolated from different donors (Figure S2). It is also worth noting that the cytokine inhibitory concentration (IC_50_) of EM-163 measured in human primary cells, which was 15 µM to 400 µM for most of the cytokines were consistent with the concentration of EM-163 that inhibited SEAP reporter activity in our earlier experiments. At IC_50_ concentration EM-163 did not show cytotoxicity in MNCs (data not shown). Cell viability (metabolically active cells) was examined by measuring ATP content using a chemiluminescence method as described elsewhere [19]. EM-163 also inhibited NF-kB p50 activation (46% to 62% at concentration 250 µM to 10 µM) in MNCs isolated from different donors treated with SEB (Figure S3). These results suggest that the dimeric BB-loop mimetic was effective in inhibiting cytokine production in human primary cells when applied either pre- or post-exposure to SEB, without affecting cellular viability.

### EM-163 Abrogates Pro-inflammatory Cytokine Response in vivo and Protects Mice when Administered Either Pre- or Post-exposure to Lethal SEB Challenge

**Figure 8:**
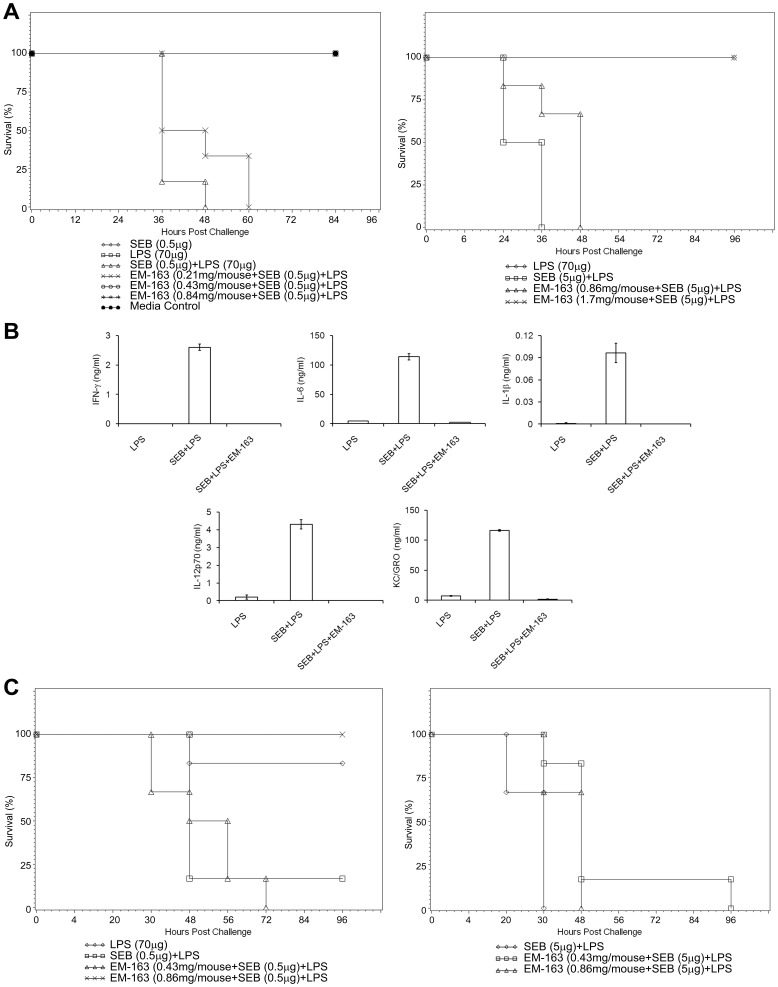
EM-163 attenuates SEB -induced pro-inflammatory serum cytokines and lethality in mice. BALB/c mice (n = 6) were injected (i.p.) with different amounts of EM-163, 30 min later injected with SEB followed by LPS 2 h later. Mice were observed to determine if mice survived, or time to death if they did not. Control mice injected only with 60 µg of LPS or 1 µg of SEB survived. Data represent three separate experiments. Kaplan Meier survival analysis and log-rank tests were performed to compare survival curves with stepdown Bonferroni adjustment for pair wise comparisons between groups. (**A)**, Pre-treatment of EM-163, 0.42 mg/mouse, or 0.86 mg/mouse protected mice against lethal SEB (0.5 µg/mouse,1 LD_50)_ challenge, p = 0.0015; Pre-treatment of EM-163 protected mice challenged with SEB (5 µg/mouse equivalent to10 LD_50),_ p≤0.0261_;_ (**B**). Administration of EM-163 in mice inhibited pro-inflammatory cytokine response. Similar to experimental settings as in Figure 7A **,** mice (n = 6) were bled at 4 h, serum were pooled from each group [LPS, SEB+LPS, EM-163 (1.7 mg/mouse)] and measured serum cytokines. (**C**). Post-exposure to SEB (0.5 µg I LD_50_), EM-163 treated mice were protected, p  = 0.0102; Post-exposure to SEB challenge, EM-163 delayed death (5 µg = 10 LD_50_), p≤0.0129.

The results presented above suggest that observed changes in signaling due to EM-163 treatment translate into a decrease in cytokine production in primary culture. We therefore examined if EM-163 treatment would likewise lead to a decrease in cytokines in a live animal and if this would protect the animals from a lethal SEB challenge. To address this, we used the LPS potentiation model of SEB toxicity in mice [5], [10], [11]. BALB/c mice (n = 6) were injected intraperitoneally (i.p.) with varying amounts of EM-163 **(**0.21 mg/mouse, 0.42 mg/mouse, and 0.86 mg/mouse) and 30 min later they were treated with SEB (0.5 µg/mouse, 1LD_50_), followed by LPS another 2 h later. All animals in the study not treated with EM-163 succumbed by 48 h. In contrast, treatment with 0.21 mg/mouse of EM-163 delayed death. Mice that were treated with EM-163 at concentrations of 0.42 mg/mouse or 0.86 mg/mouse were completely protected (p = 0.05) (Figure 8A ). When mice were exposed to a higher lethal dose of SEB (5 µg/mouse, 10 LD_50_), all mice not treated with EM-163 succumbed by 30 h. In contrast, EM-163 pre-treatment with 0.86 mg/mouse delayed death and pre-treatment with 1.7 mg/mouse protected completely [Figure 8A]. Using the same experimental paradigm as for the results shown in Figure 8A, we determined the pro-inflammatory serum cytokine profile at the 4 h time point. The results shown in Figure 8B indicated that the cytokine response was abrogated in mice treated with EM-163, suggesting that cytokine responses are correlated with the survival of the mice. In addition to pre-treatment, our results also showed that mice treated with EM-163 (0.86 mg/mouse) 30 min after SEB exposure (0.5 µg/mouse, 1LD_50_) were completely protected. With a higher challenge dose of SEB (5 µg/mouse, 10LD_50_) EM-163 still delayed the death of mice (p = 0.012, log-rank pairwise comparisons tests) (Figure 8C). As for BALB/c mice challenged with SEB, C57BL/6 mice pretreated with EM-163 and challenged with a lethal dose of SEA likewise showed inhibition of cytokine responses and were protected (Figure S4). These results suggest that EM-163 effectively inhibited pro-inflammatory cytokine responses and protected mice from toxic shock-induced death from lethal SEB or SEA challenge. Taken together, the results indicate that EM-163 has substantial therapeutic potential against SEB intoxication.

## Discussion

Our previous studies showed that MyD88-mediated pro-inflammatory cytokine signaling is critical to SEB toxicity [11], [12]. This indicated that MyD88 was a valid therapeutic target and subsequent preliminary work using a synthetic mimetic of the BB-loop of the Toll/IL-1 receptor (TIR) domain of MyD88, provided proof of concept for this strategy to treat SEB-induced toxic shock syndrome [19]. Here, we have shown that synthesis of a BB-loop mimetic with dual functional groups greatly enhanced the biological activity and target specificity of the mimetic, a large improvement over earlier work. The “dimeric” compound EM-163 attenuated TNF-α, IFN-γ, IL-1β, IL-2 and IL-6 production (IC_50_ 15–400 µM) in human primary cells when applied either pre- or post-exposure to SEB. Our biochemical and cell-based reporter assay data suggest that EM-163, by targeting MyD88, inhibits pro-inflammatory cytokine signaling. Most importantly, administration of EM-163 abrogated pro-inflammatory cytokine production and completely protected mice from the toxic shock induced-death of a lethal SEB challenge.

Recent reports suggest that MyD88-mediated pro-inflammatory cytokine signaling is not limited to the TLR/IL-1R, but rather is generally shared by other receptors such as MHC receptors [13]. The role for MHC class II molecules in TLR signaling was first suggested by studies that showed macrophages with no or low expression of MHC class II had a defective inflammatory response to LPS-stimulation [28]. It has also been reported that MHC class II molecules enhance toll-like receptor mediated innate immune responses [29]. In line with these observations, our recent results demonstrated that SEB, which binds to MHC class II receptors, activates MyD88-mediated pro-inflammatory signaling [12]. Thus, in addition to their classical function in antigen presentation, MHC class II molecules are now shown to promote MyD88 signaling. As the MHC class II molecule lacks any known signaling motifs in its short cytoplasmic tail, it has been proposed that it might use associated signaling molecules at the membrane. These signaling molecules include cell receptors (such as CD20, CD79, CD23) and members of the immunoglobulin family (such as CD19), the tetraspan family (such as CD81 and CD82), and the TNF receptor family (such as CD40). All of these receptor families have been shown to associate with MHC class II molecules, and thus may likely be involved in initiating intracellular signaling [13], [30]. Whether these signaling proteins are functionally linked, either directly or indirectly, to MHC class II molecules and adaptor protein MyD88 is a question that is currently under investigation in our laboratory. Nevertheless, results from our laboratory and others have demonstrated that TLR and MHC-mediated responses both engage the adaptor molecule MyD88 [14], [31]. Likewise, SEB engagement of MHC class II molecules has been shown to up-regulate both TLR4 [32] and MyD88 (Kissner et al. 2011, unpublished observation) on human monocytes. Previous work from our laboratory showed that LPS potentiates SEB-induced lethal shock in mice [5], [11], [12]. LPS primarily interacts with CD14 receptors on macrophages, while SEB triggers MHC class II-positive cells and T-cells to release pro-inflammatory cytokines. Therefore, it is likely that MHC class II-SEB binding up regulates MyD88 and TLR4, and subsequent LPS stimulation may synergistically trigger interaction between the respective TIR domains of MyD88 and TLR/IL-1R1 receptors. This causes activation of several signaling cascade pathways that include nuclear factor NF-kB and transcription factor AP-1, as well as various stress-associated kinases, resulting in release of pathological levels of pro-inflammatory cytokines. Although the underlying mechanism by which MHC class II signals recruit MyD88 is currently under investigation, the adaptor protein is known to integrate signals through TIR domain-TIR domain interactions [16], [33]. In this study, our results indicated that the compound EM-163 (designed to be structurally similar to the BB-loop in the TIR domain of MyD88) inhibited the MyD88-mediated signaling initiated by exposure to SEB. In addition, a preliminary result from our laboratory indicated that EM-163 was capable of inhibiting MyD88-mediated pro-inflammatory cytokine responses with exposure to *Francisella tularensis* LPS or irradiated *Burkholderia mallei* (unpublished observation). As signaling through MyD88 appears to be a common link in many of the host-directed inflammatory process, it is likely that EM-163 may have a potential for broader use against exposure to other bio-threat agents.

The most potent microbial products implicated in the pathogenesis of septic shock are gram-positive-derived superantigenic exotoxins and gram-negative endotoxins. Both types of toxin induce comparable pro-inflammatory cytokines from human mononuclear cells *in vitro*, cause lethal shock *in vivo*, and have been identified in the bloodstream of critically ill patients [8], [9]. The combination of endotoxins and superantigens has been shown to have particularly severe consequences in several animal models. For example, it has been reported that SEB potentiates LPS-induced hepatic dysfunction and cytokine responses in chronically catheterized rats [34]. Results from our laboratory also firmly established that LPS and SEB synergize to produce a dose dependent toxicity in mice that is several fold higher than the toxicity of SEB or LPS alone. Our results raise the possibility that recognition of SEB by MHC class II receptors may exacerbate the pro-inflammatory response of monocytes to gram-negative infection or endotoxin through activation of a common MyD88-mediated signaling. In both cases, targeting of MyD88 by EM-163 inhibited pro-inflammatory cytokine signaling and protected mice from SEB intoxication. EM-163 treatment caused intracellular accumulation of MyD88 in primary monocytes when stimulated with SEB. It is likely that EM-163 binds to MyD88, including MyD88 that is newly synthesized in response to SEB, rendering it incapable of downstream signal transduction. EM-163 may or may not be capable of disrupting dimers of the MyD88 TIR domain, but it is clear that stable, monomeric forms of MyD88 must exist since they engage interchangeably in both homo and heterotypic interactions. It seems likely that the monomeric form of the MyD88 TIR domain, with its exposed BB loop, is the molecular target of EM-163.

In summary, our results provide evidence that a synthetic BB-loop mimetic, EM-163, that targets the TIR-domain of MyD88 can limit hyper-inflammation and prevent toxic shock. The effectiveness of this mimetic when administered either pre- or post-SEB exposure further supports its therapeutic potential. An ongoing effort is underway to further refine EM-163 by chemical modification to increase potency, bioavailability and drug-like properties for potential clinical use against toxic shock.

## Supporting Information

Figure S1 **EM-163 targets newly expressed MyD88.** MyD88 KO HEK293 (HEK293-I3A) cell line were co-transfected with plasmids MyD88 flag and pCMV-HA-MyD88. Seven hours after transfection, cells were incubated for 13 h with or without EM-163 (100 µM to 1 µM). At the end of incubation, cells were lysed, and cytoplasmic fractions were separated. (**A**), MyD88 expression was detected by Western blot analysis using anti-MyD88 antibody. (**B**), Cell extracts were immunoprecipitated (IP) with anti-Flag antibody, and immune-precipitated proteins were analyzed in Western blot with anti-MyD88 antibody. (**C**) Immunoblot (B) was stripped and reprobed with anti-HA antibody. The results are representative of two independent experiments.
(TIF)

Figure S2 **EM-163 inhibits SEB-induced cytokine response in different donors**. MNCs (1×10^6^) from two normal donors (Donor 1 and Donor 2) were cultured with SEB (200 ng/ml) with or without EM-163 (500 µM to 10 µM) for 20 h. The culture supernatants were collected and measured for cytokine by MSD assay. The IC_50_ value was calculated as the concentration required for inhibition of cytokine production by 50% relative to the control. Data are representative of three experiments.
(TIF)

Figure S3 **Inhibition of NF-kB p50 activation in the presence of EM-163 in MNCs stimulated with SEB**. Activation of NF-kB in primary mononuclear cells treated with SEB in the absence or presence of different concentration EM-163 was determined as described elsewhere [12]. Data are presented in the figure as percentage increase over the control and represent one of three experiments using separate donors. Significant differences (p≤0.005) are indicated for MNCs treated with SEB *vs* MNCs treated SEB in the presence of EM-163 (*).
(TIF)

Figure S4 **EM-163 attenuates pro-inflammatory cytokines and SEA induced lethality in mice.** (**A**)**,** Administration of EM-163 protected mice from toxic shock induced death challenged with lethal dose of SEA. C57BL/6 mice mice (n = 4) were injected with EM-163 (0.85 mg, or 1.7 mg, 100 µl volume/mouse), 30 min later injected with SEB (5 µg/mouse) followed by LPS 2 h later. Mice were observed for survival. Control mice injected with 150 µg of LPS or 1 µg of SEB survived. Data are representative of two separate experiments; (**B**), Administration of EM-163 in mice inhibited pro-inflammatory cytokine response**,** mice were bled at 24 h, serum were pooled from each group and measured serum cytokines.
(TIF)
