# Supplementary figures and images for: Therapeutic Inhibition of Pro-Inflammatory Signaling and Toxicity to Staphylococcal Enterotoxin B by a Synthetic Dimeric BB-Loop Mimetic of MyD88
Source: PLoS One. 2012 Jul 27;7(7):e40773. doi: 10.1371/journal.pone.0040773 (PMC3407147; doi:10.1371/journal.pone.0040773)

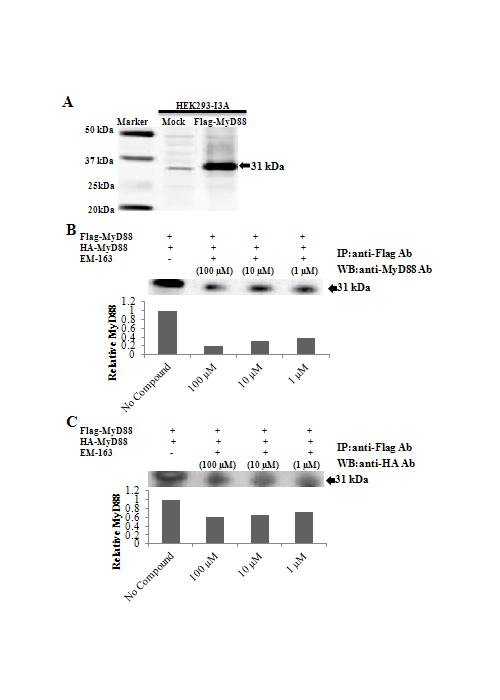

Supplement: Figure S1 — EM-163 targets newly expressed MyD88. MyD88 KO HEK293 (HEK293-I3A) cell line were co-transfected with plasmids MyD88 flag and pCMV-HA-MyD88. Seven hours after transfection, cells were incubated for 13 h with or without EM-163 (100 µM to 1 µM). At the end of incubation, cells were lysed, and cytoplasmic fractions were separated. (A), MyD88 expression was detected by Western blot analysis using anti-MyD88 antibody. (B), Cell extracts were immunoprecipitated (IP) with anti-Flag antibody, and immune-precipitated proteins were analyzed in Western blot with anti-MyD88 antibody. (C) Immunoblot (B) was stripped and reprobed with anti-HA antibody. The results are representative of two independent experiments. (TIF) [file pone.0040773.s001.tif]

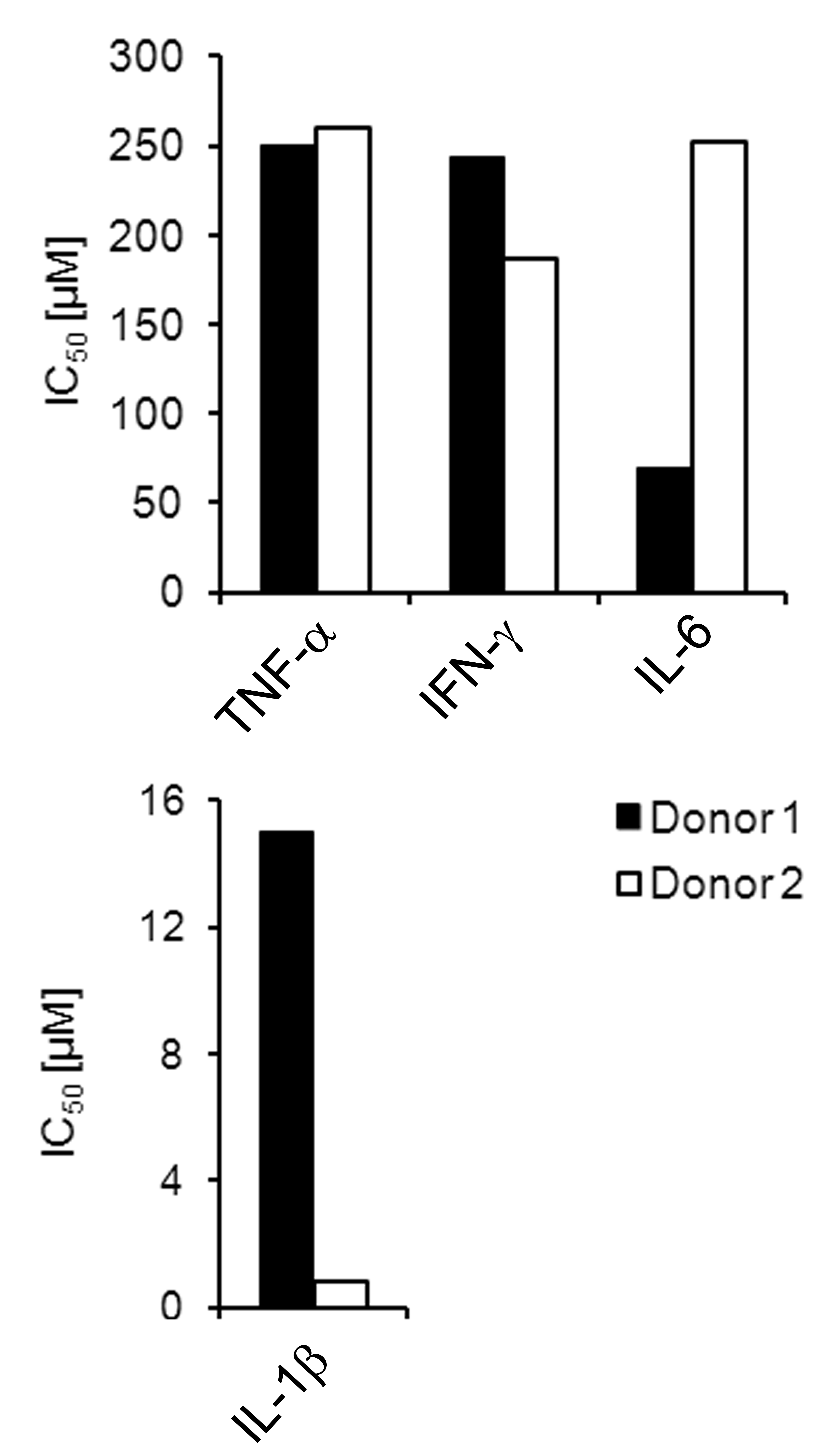

Supplement: Figure S2 — EM-163 inhibits SEB-induced cytokine response in different donors. MNCs (1×10^6^) from two normal donors (Donor 1 and Donor 2) were cultured with SEB (200 ng/ml) with or without EM-163 (500 µM to 10 µM) for 20 h. The culture supernatants were collected and measured for cytokine by MSD assay. The IC50 value was calculated as the concentration required for inhibition of cytokine production by 50% relative to the control. Data are representative of three experiments. (TIF) [file pone.0040773.s002.tif]

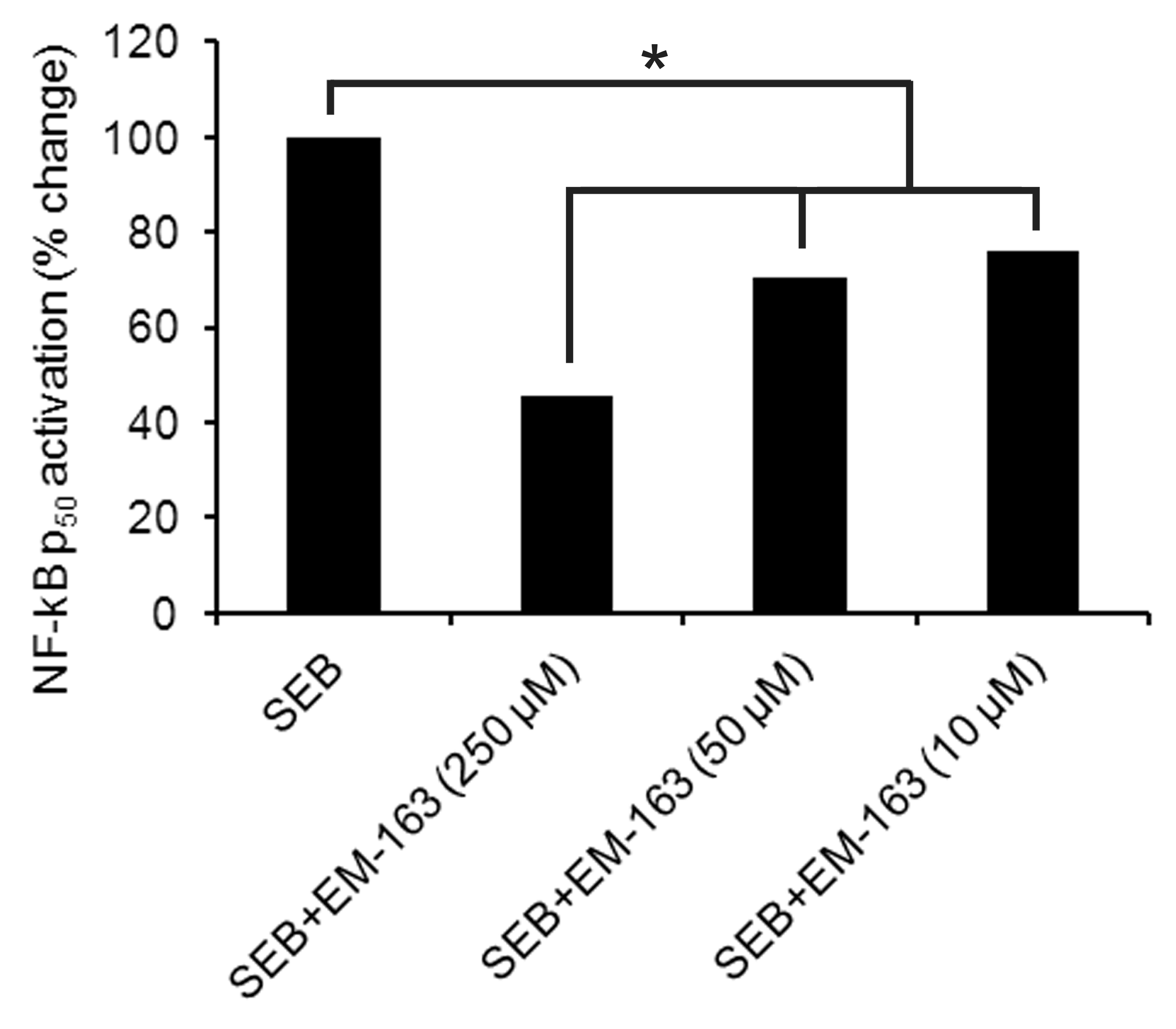

Supplement: Figure S3 — Inhibition of NF-kB p50 activation in the presence of EM-163 in MNCs stimulated with SEB. Activation of NF-kB in primary mononuclear cells treated with SEB in the absence or presence of different concentration EM-163 was determined as described elsewhere [12]. Data are presented in the figure as percentage increase over the control and represent one of three experiments using separate donors. Significant differences (p≤0.005) are indicated for MNCs treated with SEB vs MNCs treated SEB in the presence of EM-163 (*). (TIF) [file pone.0040773.s003.tif]

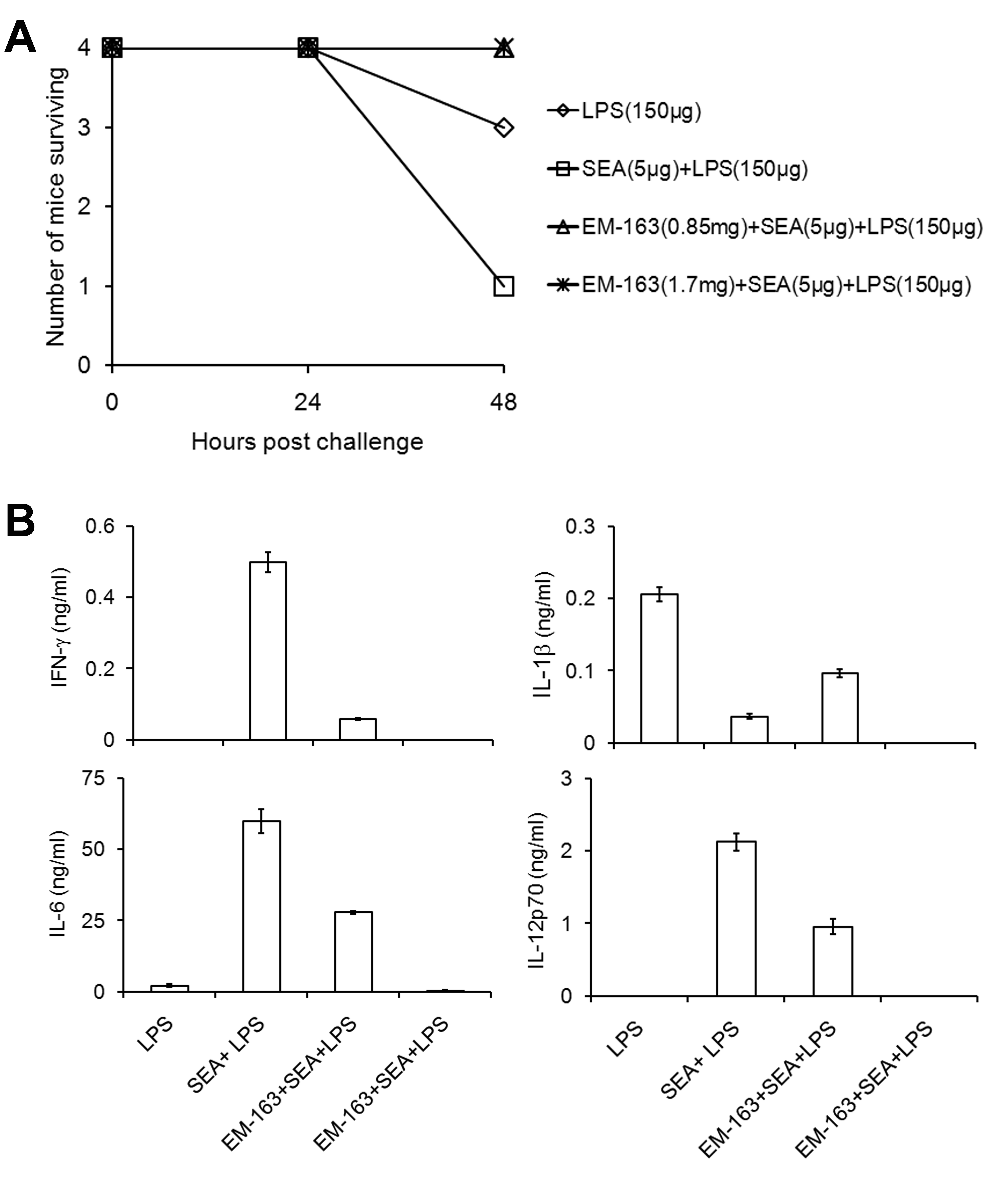

Supplement: Figure S4 — EM-163 attenuates pro-inflammatory cytokines and SEA induced lethality in mice. (A), Administration of EM-163 protected mice from toxic shock induced death challenged with lethal dose of SEA. C57BL/6 mice mice (n = 4) were injected with EM-163 (0.85 mg, or 1.7 mg, 100 µl volume/mouse), 30 min later injected with SEB (5 µg/mouse) followed by LPS 2 h later. Mice were observed for survival. Control mice injected with 150 µg of LPS or 1 µg of SEB survived. Data are representative of two separate experiments; (B), Administration of EM-163 in mice inhibited pro-inflammatory cytokine response, mice were bled at 24 h, serum were pooled from each group and measured serum cytokines. (TIF) [file pone.0040773.s004.tif]
